# Inflammatory processes and elevated osteoclast activity chaperon atrophic non-union establishment in a murine model

**DOI:** 10.1186/s12967-019-02171-4

**Published:** 2019-12-12

**Authors:** Johannes M. Wagner, Sonja V. Schmidt, Mehran Dadras, Julika Huber, Christoph Wallner, Stephanie Dittfeld, Mustafa Becerikli, Henriette Jaurich, Felix Reinkemeier, Marius Drysch, Marcus Lehnhardt, Björn Behr

**Affiliations:** University Hospital BG Bergmannsheil Bochum, Bürkle-de-la-Camp Platz 1, 44789 Bochum, Germany

**Keywords:** Atrophic non-union, Osteoclasts, Bone regeneration, Osteoimmunology

## Abstract

**Background:**

Delayed bone healing, especially in long bones poses one of the biggest problems in orthopeadic and reconstructive surgery and causes tremendous costs every year. There is a need for exploring the causes in order to find an adequate therapy. Earlier investigations of human scaphoid non-union revealed an elevated osteoclast activity, accompanied by upregulated levels of TGF-beta and RANKL. Interestingly, scaphoid non-union seemed to be well vascularized.

**Methods:**

In the current study, we used a murine femur-defect model to study atrophic non unions over a time-course of 10 weeks. Different time points were chosen, to gather insights into the dynamic processes of non-union establishment.

**Results:**

Histological analyses as well as western blots and qRT-PCR indicated enhanced osteoclast activity throughout the observation period, paralleled by elevated levels of TGF-beta, TNF-alpha, MMP9, MMP13 and RANKL, especially during the early phases of non-union establishment. Interestingly, elevated levels of these mediators decreased markedly over a period of 10 weeks, as inflammatory reaction during non-union establishment seemed to wear out. To our surprise, osteoblastogenesis seemed to be unaffected during early stages of non-union establishment.

**Conclusion:**

Taken together, we gained first insights into the establishment process of atrophic non unions, in which inflammatory processes accompanied by highly elevated osteoclast activity seem to play a leading role.

## Background

Although bone regeneration is a reliable process, fracture healing is often accompanied with a variety of complications. One of the most common problems is non-union establishment after a delayed healing period [1]. For instance, the rate of non-unions after open tibial fractures is considered around 17% [2]. Non-unions can be defined as either atrophic or hypertrophic based on radiological aspects [3]. While hypertrophic non-unions constitute normal callus tissue without bony bridging, atrophic non-unions show no relevant callus tissue. Furthermore, hypertrophic non-unions are mostly caused by a lack of fracture stability, while causes for atrophic non-unions are quite diverse [1]. There are several risk factors that contribute to atrophic non-union, such as gender, age, diabetes, osteoporosis, smoking and NSAIDs [4]. However, surprisingly little is known about their exact causes [4–6]. Novel therapies like extracorporal shock wave therapy could show improvement targeting hypertrophic non-unions, but are less effective for atrophic non-unions [7]. For instance, it was proposed that impaired vascularization is a critical key factor for delayed bone healing [8]. Previously, an avascular fracture site was contemplated to be causative for atrophic non-union establishment [9, 10]. This hypothesis was maintained by different authors, who could demonstrate impaired fracture healing by virtue of inhibiting angiogenesis during fracture healing in animal models [11–13]. Conversely, stimulation of angiogenesis could improve impaired fracture healing [14–16]. In contrast to these findings, other authors showed that atrophic non-unions seem to be well vascularized [17–20]. In a murine animal model, Garcia et al. [21] observed no difference in vessel density and increased levels of VEGF-A in the non-union group at early and late time points upon atrophic non-union establishment. Accordingly, in our own work we could find human scaphoid non-unions to be well vascularized. Summarized, the exact role of angiogenesis in atrophic non-union establishment is still subject to debate.

Additional research focused on the role of BMPs and other factors critical for bone regeneration or atrophic non-union establishment [22–25].

Thus, reasons for atrophic non-union establishment still remain unclear and inflammatory processes with resulting osteoclastogenesis have not been investigated in detail.

Recent data from our investigation on established human atrophic scaphoid non-unions revealed that gene-expression of TGF-β, Wnt 5a and RANKL were significantly upregulated as compared to healthy bone tissue [21]. To our surprise, results of this study showed that inflammatory processes and subsequently osteoclastogenesis could play a key role.

Even though human data can be considered ideal when tackling such an important problem, the fact that we were restricted to analyze specimens of constituted non-unions left us with an unsolved problem: exploring mechanisms and dynamics that contribute to atrophic non-union establishment in this setting is unfeasible. Therefore, the study presented here approached this paucity and provided a unique opportunity to translate the results we obtained from human samples into an animal model. As the dynamic processes of non-union establishment are more or less unclear, this study aimed to reveal them in an established murine model of atrophic non-unions.

Therefore, we adapted an existing murine animal model, generating an atrophic non-union in mouse femur [26].

## Methods

### Surgical procedure

12 week old C57BL/6J male and female mice with an average weight of 25 g were used for this project. Surgical steps were performed, as previously described [26].

In preparation of surgical procedure, the right leg was shaved and disinfected. A 4 mm medial parapatellar incision was placed and the patella was dislocated laterally. Then a 0.5 mm hole was drilled into the intracondylar notch of the femur and a distally flattened 24G needle was implanted intramedullary into the right femur of the animal. Then the implanted pin was flattened at the distal end to prevent secondary pin loss. Then the patella was replaced and the wound was closed. After implantation of the pin a second incision was made in the middle of the lateral thigh. The musculus quadriceps was lateralized and the femur was exposed. Afterwards, a custom-made clip of 6 mm length was implanted into the ventro-dorsal femur. Then an osteotomy with a gap size of 1.8 mm was created in the non-union group with a fine drilling device [proxxon micromot 50, proxxon, Trier (Germany)] between the metallic clip using size-standardized spherical trephines under permanent saline cooling to prevent thermal damage around the defect site. In the control group a gap size of 0.25 mm was created accordingly with a Gigli saw [Gigli saw wire, 0,25 mm, AO Research Institute, Davos (Switzerland)]. The small gap size of 0.25 mm in the control group was mimicking physiological bone regeneration after bone fractures. The metallic clip inserted before guaranteed maintenance of the gap size. The gap sizes was controlled by custom-made template of 1.8 mm. The periosteum of the fracture site was left intact. Afterwards the wound was closed.

### Histology, immunohistochemistry and histomorphometry

Data presented are a representative of at least three independent experiments.

At the indicated time points (48 h and 1, 5, 10 weeks) femora of six animals per group were harvested, the metallic pin and clip removed and fixed in 4% paraformaldehyde overnight, decalcified in 19% EDTA and paraffin embedded. Femora were then longitudinally sectioned at 9 µm on a Microtome. Tartrate-resistant acid phosphatase (TRAP) staining, which is a reliable marker detecting mature osteoclasts, was performed with TRAP Kit (Sigma-Aldrich, St. Louis, USA) after manufacturers instruction.

Additionally, immunohistochemical staining with primary antibodies against Osteocalcin and PCNA (Santa Cruz Biotechnologies, Dallas, USA) were performed using Vectastain ABC Kit (Vector Laboratories, Burlingame, USA). After deparaffinization and rehydration, specimens were incubated with Proteinase K for antigen demasking. Endogenous peroxidase activity was quenched by incubation with 3% hydrogen peroxide solution. Thereafter, specimens were blocked with normal blocking serum to prevent unspecific binding of primary antibody that was subsequently applicated and incubated overnight at 4 °C. Following, secondary antibody conjugated to horseradish peroxidase (HRP) was used and staining reaction was performed by use of NovaRED (HRP) Peroxidase Substrate Kit (Vector Laboratories, Burlingame, USA).

For immunofluorescent staining, primary antibody against RUNX2 (Santa Cruz Biotechnologies, Dallas, USA) was used. Initial steps were carried out similar to immunohistochemical staining until application of primary antibody. Thereafter, samples were incubated with secondary antibody conjugated to Alexa Fluor594 (Thermo Fisher Scientific, Waltham, USA). Images were taken with Zeiss Axioplan microscope.

Histomorphometry of immunohistochemical staining was performed with Photoshop (Adobe, San Jose, USA). Pixels were counted semiautomatically using the magic wand tool (tolerance 60, noncontiguous).

### RNA isolation and gene expression analysis

For RNA isolation, femoral defects of four animals per group were homogenized and RNeasy mini kit was used according to the manufacturer’s instruction (Qiagen, Hilden, Germany). Quantitative real-time polymerase chain reaction (qRT-PCR) was performed using TaqMan probes in StepOnePlus (Applied Biosystems, Foster City, USA) performed in triplicates. Expression data was normalized with housekeeping genes (Gapdh and 18S) and calculated with the ∆Ct method. Genes examined were osteogenesis-related genes TGF-β, Runx2, and osteoclastogenesis-related genes RANKL, OPG and DCSTAMP. Moreover, inflammation-related genes TNF-α, MMP 9, 13 were analyzed. Primer sequences have been obtained with Primer-Blast (NIH).

### Western Blot analysis

Isolated protein of 6 animals per group was combined and mixed with Laemmli sample buffer. After denaturation at 95 °C, samples were kept on ice until loading of the SDS PAGE. 15% polyacrylamide gels were used for electrophoresis of 30 µg total protein per lane. Protein was transferred to a nitrocellulose membrane using wet transfer method before membranes were blocked with 3% bovine serum albumin to prevent unspecific binding. After washing, membranes were incubated with primary antibodies RANKL, Runx2, TNF-α, TGF-β and MMP13 (Abcam, Cambridge, UK) overnight at 4 °C, followed by washing and incubation with HRP-conjugated secondary antibody (Thermo Fisher Scientific, Waltham, USA; Santa Cruz Biotechnologies, Dallas, USA). Proteins were detected for 30–60 s by enhanced chemoluminescence.

### Statistics

Results are presented as mean ± standard error of the mean (SEM), while p-values were calculated by student’s t-test comparing two groups and ANOVA comparing more than two groups. Tukey’s test was used for post hoc comparisons. Statistical significances were set at a p-value < 0.05.

## Results

### Osteoblast and osteoclast function during non-union establishment

**Fig. 1:**
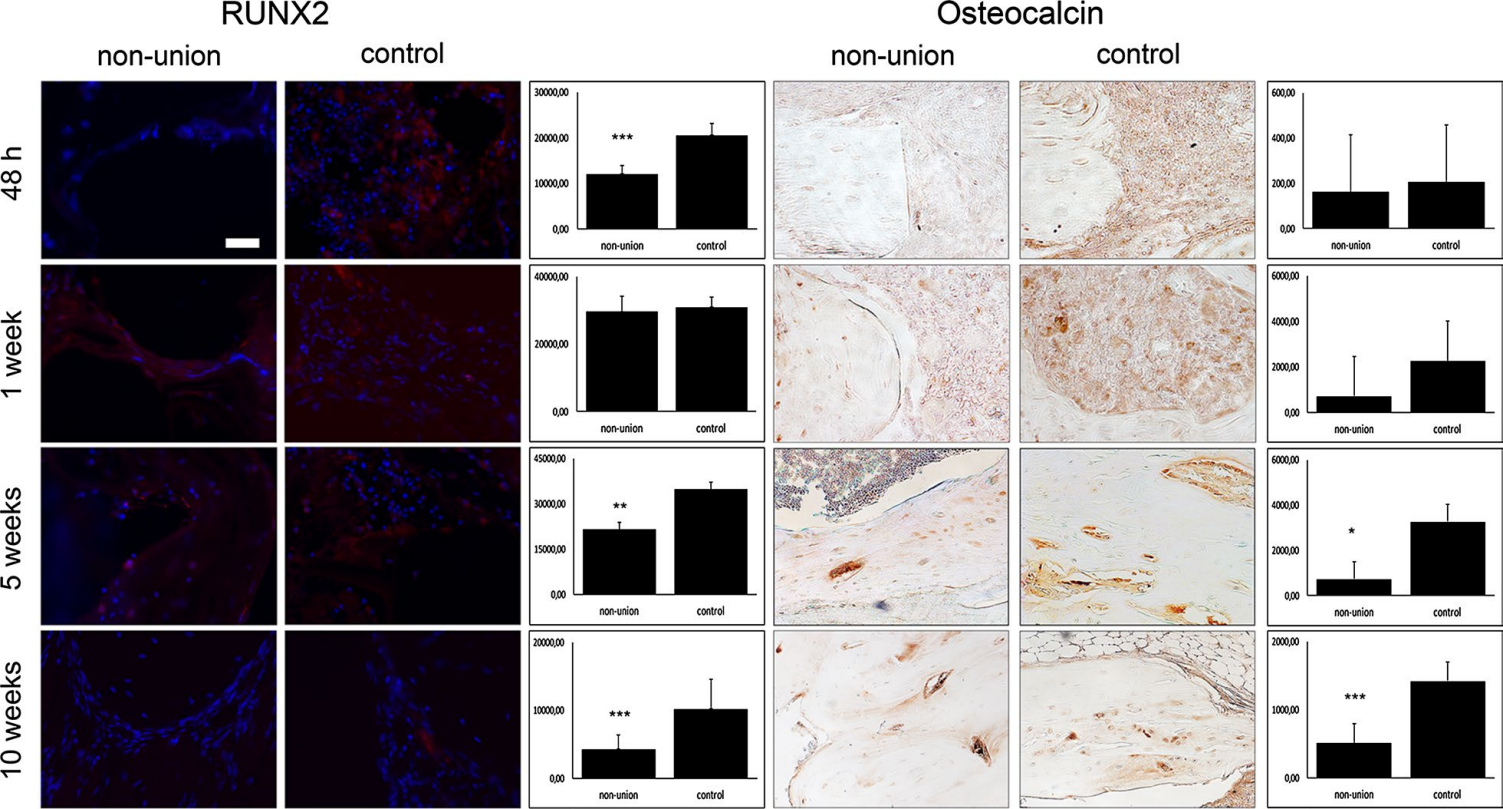
Osteoblasts are unaltered at early non-union establishment. Immunoflourescent stainings against Runx2 and Osteocalcin of non-union and control. Surprisingly, stainings evaluating osteoblastogenesis (Runx2) and osteoblast maturation (osteocalcin) revealed an unaltered osteoblast function at early non-union establishment processes, ranging 1 week after surgery. However, Runx2 and Osteocalcin stainings showed less signaling at 48 h and 5 and 10 weeks, indicating reduced osteoblasts at this time points. Scale bar represents 100 µm; p-value: *< 0.05

**Fig. 2:**
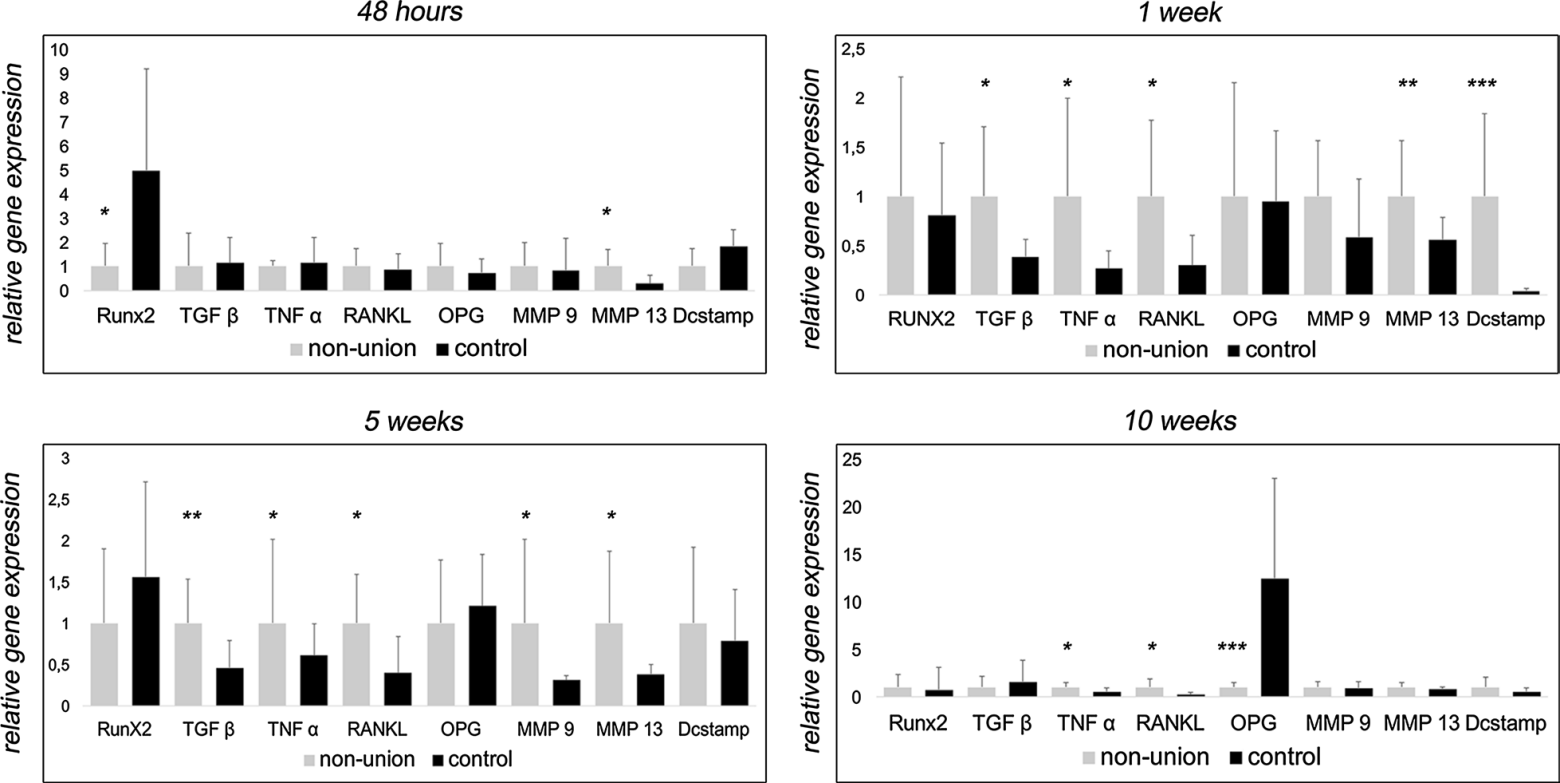
Inflammatory mediators contribute to non-union establishment. QRT-PCR of osteogenesis-related genes Runx2, TGF-β, osteoclastogenesis-related genes RANKL, OPG and Dcstamp and inflammatory markers TNF-α, MMP9 and MMP13. According to stainings, Runx2 levels are only impaired at early fracture healing. Interestingly, while RANKL levels are elevated throughout non-union establishment, OPG expression seems to be elevated to physiological levels of fracture healing, except for established non-union after 10 weeks. Surprisingly, low levels of Dcstamp, important for osteoclast fusion, were detected in non-union and control except for the first week after surgery, indicating excessive osteoclast formation. Inflammatory markers TNF-α, MMP9 and MMP13 as well as TGF-β showed to be enhanced 1 and 5 weeks after surgery. p-value: *< 0.05

**Fig. 3:**
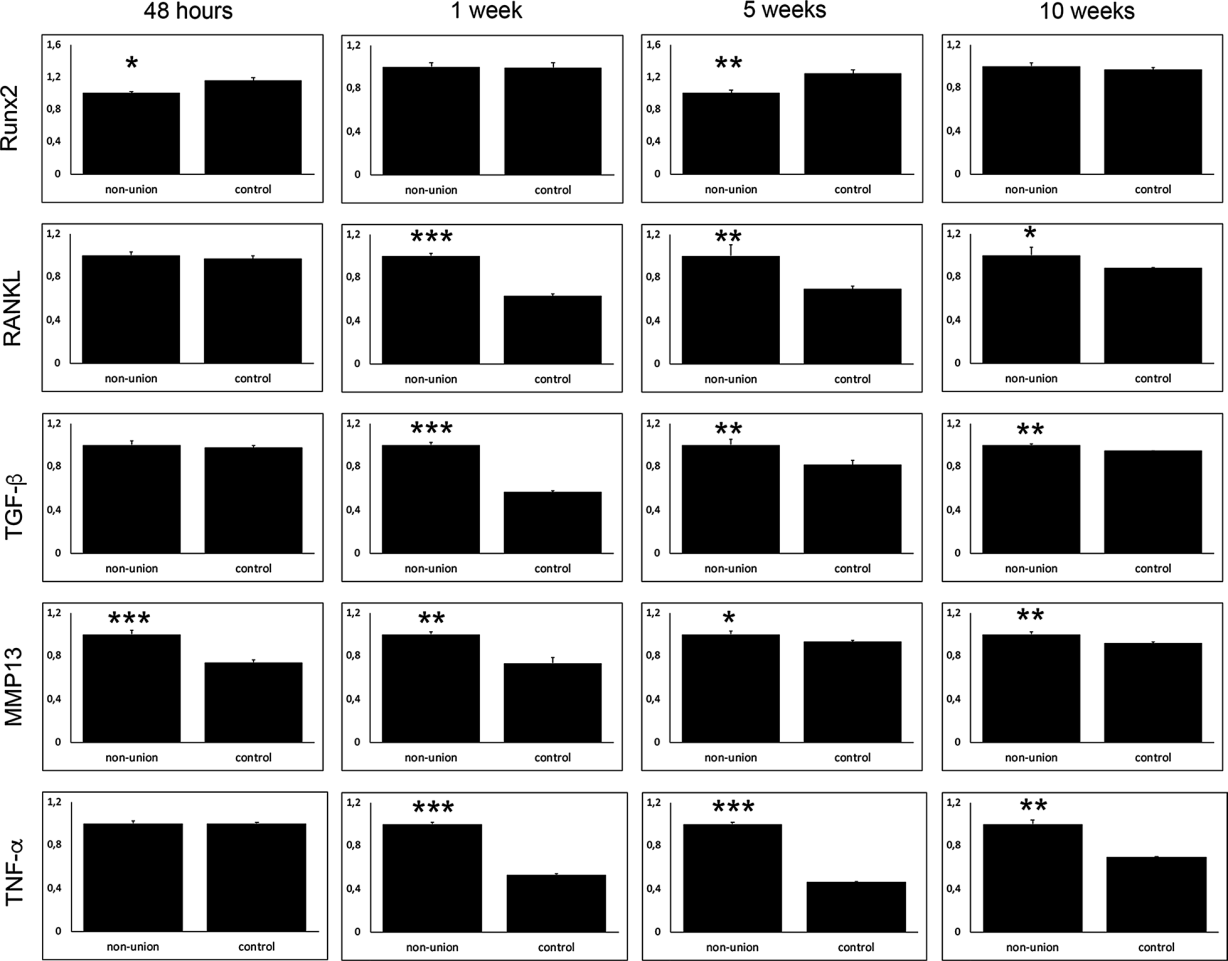
Western blot verifies inflammatory reaction during non-union establishment. Western blots against Runx2, TNF-α, MMP13, RANKL and TGF-β. According to qRT-PCR results protein levels of Runx2 in non-unions seemed to be similar to control, except for early fracture healing, indicating a physiological osteoblastogenesis during non-union establishment. Moreover, RANKL mediated Osteoclast activity seemed to be upregulated during the whole non-union establishment process. This was accompanied by an inflammatory reaction, including TNF-α, MMP13 and TGF-β as central mediators. p-value: *< 0.05; ** < 0.01; ***< 0.001

Our primary interest investigating the reasons for non-union establishment was to evaluate osteoblast and osteoclast function and reveal dysregulated bone homeostasis of formation and resorption. Osteoblastogenesis evaluated in runt-related transcription factor 2 (Runx2) staining, gene expression analysis and western blot showed significant downregulation at 48 h, 5 weeks and 10 weeks, compared to control group (Figs. 1, 2, 3). Mature osteoblasts detected by osteocalcin staining were significantly diminished in the non-union group at 5 and 10 weeks (Fig. 1).

**Fig. 4:**
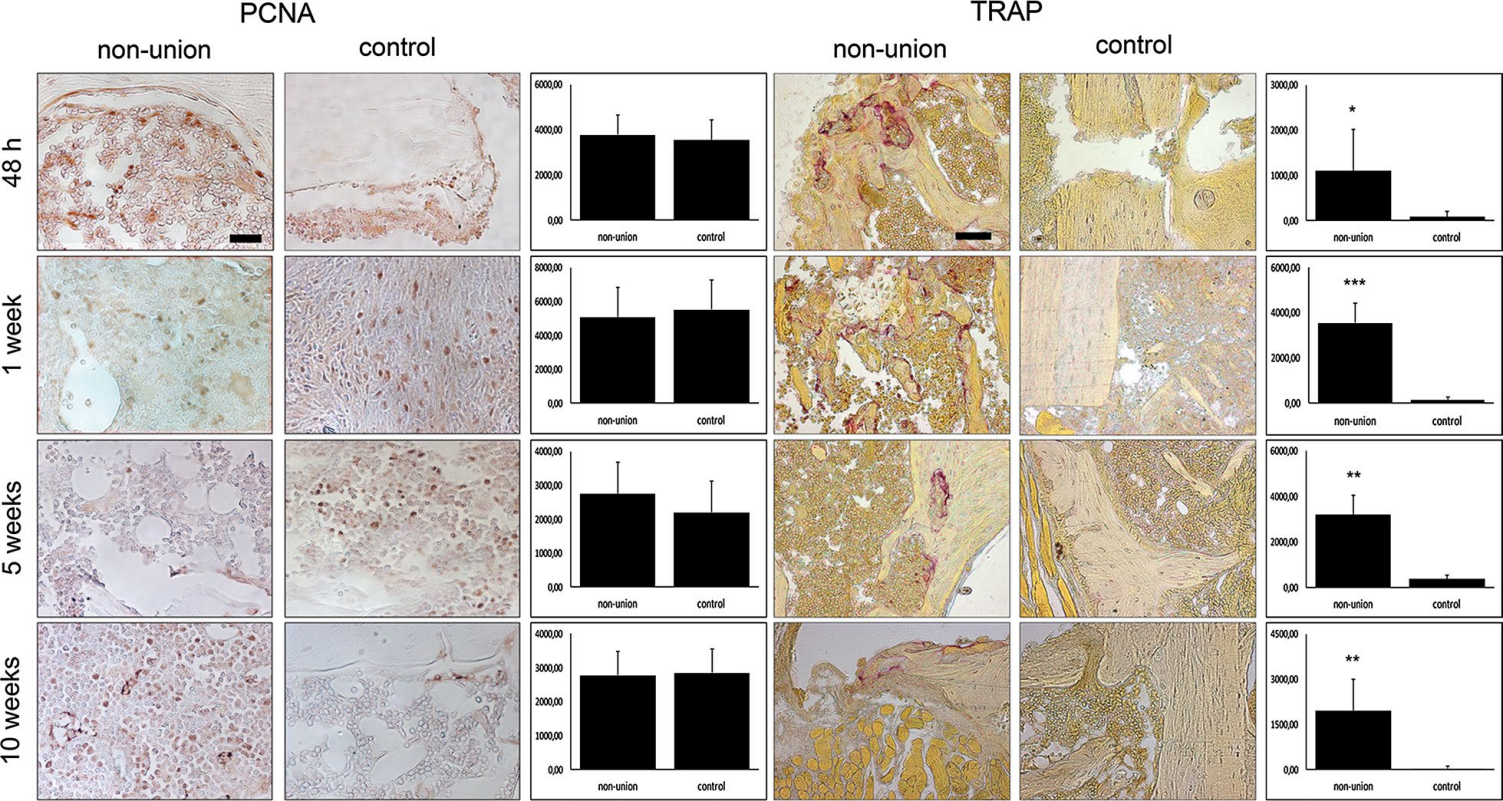
Elevated osteoclast activity in non-unions comes with high proliferative activity. Immunohistochemical staining with primary antibody against PCNA and TRAP-staining. Elevated RANKL levels in non-unions, which could be seen in qRT-PCR and western blots lead to excessive osteoclast activity, spotted in TRAP-stainings. Interestingly, cell proliferation in non-unions evaluated by PCNA stainings was highly activated just similar to control animals. Scale bar represents 100 µm; p-value: *< 0.05; **< 0.01; ***< 0.001

Osteoclast number, which could be measured in TRAP staining, was highly elevated throughout the whole observation period in non-unions (Fig. 4). Interestingly, cell proliferation levels state, that the two groups were equal at all time points, seen in proliferating cell nuclear antigen (PCNA) staining (Fig. 4).

### Elevated cytokine levels in non-union establishment reveal an inflammatory reaction

Non-union establishment appeared to undergo an inflammatory reaction which was mediated by several cytokines. Levels of transforming growth factor β (TGF-β), tumor necrosis factor α (TNF-α), matrix metallopeptidase 9 (MMP9) and MMP13 showed to be increased after 1 and 5 weeks in non-union group validated in qRT PCR and Western Blot (Figs. 2 and 3).

### Osteoclast activity is elevated via RANKL/OPG-axis

According to elevated osteoclast activity, levels of RANKL were upregulated in qRT-PCR and western blots throughout observation period, except for 48 h (see Figs. 2 and 3). Interestingly, in non-unions osteoprotegerin (OPG), a well-known decoy receptor for RANKL, showed comparable levels to control till 10 weeks after surgery, when OPG levels dropped markedly in non-unions. Moreover, dendrocyte expressed seven transmembrane protein (Dcstamp), important for osteoclastogenesis was highly elevated in non-unions 1 week after surgery.

## Discussion

We observed upregulated levels of RANKL consistent with elevated osteoclast activity at all time points analysed in our murine model of non-union establishment. Levels of TGF-β, TNF-α and MMP13 were also upregulated one and 5 weeks after surgery and subsequently seemed to have important regulatory functions in this context.

The findings of this study have identified a number of different molecular targets, which may be useful for future research and novel therapeutic approaches for preventing delayed bone healing and non-union.

Interestingly, investigating the bone homeostasis of bone formation and resorption, we observed a physiological osteoblastogenesis during the early stages of fracture healing in the non-union model. However, in established non-unions, 10 weeks after surgery, osteoblasts were markedly downregulated. Consistent to this finding, cell proliferation was comparable to control, indicating non-union establishment as a highly active process.

It is well accepted that osteoblast function is impaired in established atrophic non-unions [27]. However, the fact that non-unions display a physiological osteoblastogenesis during early non-union establishment seemed striking. Thus, the unaffected bone formation seemed not to have the capability to overcome elevated bone resorption in non-union establishment as RANKL-mediated osteoclast activity was highly increased during the whole process. Continuative exploration of the exact regulation of osteoclasts, the leading role of RANKL/OPG-axis became evident during our studies. OPG is the main antagonist to osteoclast-activating RANKL [28]. Although it seemed striking that OPG levels in non-unions after 10 weeks dropped markedly with an exaggerated fold change between non-unions and control we assume a compensatory mechanism against upregulated RANKL during non-union establishment. Accordingly, dendrocyte expressed seven transmembrane protein (DC-STAMP) which is essential for osteoclast fusion and maturation [29] was enhanced during non-union establishment.

Consistent to our findings, Lienau et al. [24] observed unaltered expression levels of Runx2 during early fracture healing in a sheep osteotomy model mimicking delayed bone healing. Moreover, excessive osteoclast activity as a substantial part of non-union establishment could already be shown in animal and human studies [21, 24, 30].

Besides elucidating the homeostasis of bone formation and resorption processes, we further emphasized to characterize the role of inflammatory agents in non-unions. Selected factors which appeared to have a major impact on non-union establishment are TNF-α, TGF-β, MMP9 and MMP13.

Interestingly, both TNF-α and TGF-β had beneficial effects on fracture healing at early stages promoting osteogenesis and osteoblastogenesis [31–33]. However, studies in the field of osteoimmunology have revealed that chronic inflammation accompanied and mediated by upregulation of TNF-α lead to delayed bone regeneration and to an increased bone destruction by increasing osteoclast activity [34–37]. Additionally, in other chronic diseases like diabetes mellitus, delayed bone healing correlated with elevated levels of TNF-α, RANKL and high osteoclast activity [38]. Investigations in the field of Rheumatoid Arthritis and other degenerative bone diseases describe a special role for TNF-α in the interplay of bone destruction upon chronic inflammation [39–41]. In this context, common treatment strategies involving modulation of TNF-α proved to be quite effective against bone resorption in rheumatoid arthritis [42].

Moreover, enhanced expression of TGF-β lead to sustained differentiation of fibroblasts and could thereby dense persisting fibrous tissue in the fracture gap [43]. Accordingly, investigations of serum from patients suffering from atrophic non-unions showed enhanced levels of TGF-β [44]. Furthermore, enhanced levels of TGF-β could stimulate MMP13 expression in osteoblasts in vitro [45] and TGF-β-stimulated MMP13 expression seemed to be involved in the pathogenesis of osteoarthritis [46].

In fact, regarding MMP9 and MMP13 a dual role in fracture healing is evident. On the one hand, both factors are crucial for cartilage resorption during physiological bone regeneration, while knockout models of MMP9 and 13 showed delayed fracture healing [47, 48]. On the other hand, overexpression of MMP13 seems to be involved in different bone resorbing diseases like osteoarthritis [46] and formation of osteolytic lesions due to multiple myeloma [49] and breast cancer [50].

## Conclusions

In summary, this novel data provides encouraging insights into the establishment of atrophic non-union. Interestingly, we could show bone resorption and elevated osteoclast activity may play an important role in non-union establishment. Furthermore, we identified numerous inflammatory agents; further studies testing new treatment strategies are under way. These studies may provide effective therapeutic approaches that prevent non-union establishment in the future.

## Data Availability

The datasets used and/or analysed during the current study are available from the corresponding author on reasonable request.

## Abbreviations

VEGF-A: vascular endothelial growth factor A
BMP: bone morphogenetic protein
TGF-β: transforming growth factor beta
RANKL: receptor activator of NF-κB ligand
OPG: osteoprotegerin
Runx 2: runt-related transcription factor 2
PCNA: proliferating-cell-nuclear-antigen
TNF-α: tumor necrosis factor alpha
DCSTAMP: dendrocyte expressed seven transmembrane protein
MMP: matrix metallopeptidase
